# Molecular Mechanisms of Mitotane Action in Adrenocortical Cancer Based on In Vitro Studies

**DOI:** 10.3390/cancers13215255

**Published:** 2021-10-20

**Authors:** Marco Lo Iacono, Soraya Puglisi, Paola Perotti, Laura Saba, Jessica Petiti, Claudia Giachino, Giuseppe Reimondo, Massimo Terzolo

**Affiliations:** Department of Clinical and Biological Sciences, San Luigi Gonzaga Hospital, University of Turin, Orbassano, 10043 Turin, Italy; marco.loiacono@unito.it (M.L.I.); paola.perotti@unito.it (P.P.); laura.saba@unito.it (L.S.); jessica.petiti@unito.it (J.P.); claudia.giachino@unito.it (C.G.); giuseppe.reimondo@unito.it (G.R.); massimo.terzolo@unito.it (M.T.)

**Keywords:** mitotane, adrenocortical carcinoma, H295 strains

## Abstract

**Simple Summary:**

Mitotane is the only approved drug for the treatment of advanced adrenocortical carcinoma and for postoperative adjuvant therapy. It is known that mitotane destroys the adrenal cortex impairing steroidogenesis, although its exact molecular mechanism is still unclear. However, confounding factors affecting in vitro experiments could reduce the relevance of the studies. In this review, we explore in vitro studies on mitotane effects, highlighting how different experimental conditions might contribute to the controversial findings. On this basis, it may be necessary to re-evaluate the experiments taking into account their potential confounding factors such as cell strains, culture serum, lipoprotein concentration, and culture passages, which could hide important molecular results. As a consequence, the identification of novel pharmacological molecular pathways might be used in the future to implement personalized therapy, maximizing the benefit of mitotane treatment while minimizing its toxicity.

**Abstract:**

Mitotane is the only approved drug for the treatment of advanced adrenocortical carcinoma and is increasingly used for postoperative adjuvant therapy. Mitotane action involves the deregulation of cytochromes P450 enzymes, depolarization of mitochondrial membranes, and accumulation of free cholesterol, leading to cell death. Although it is known that mitotane destroys the adrenal cortex and impairs steroidogenesis, its exact mechanism of action is still unclear. The most used cell models are H295-derived cell strains and SW13 cell lines. The diverging results obtained in presumably identical cell lines highlight the need for a stable in vitro model and/or a standard methodology to perform experiments on H295 strains. The presence of several enzymatic targets responsive to mitotane in mitochondria and mitochondria-associated membranes causes progressive alteration in mitochondrial structure when cells were exposed to mitotane. Confounding factors of culture affecting in vitro experiments could reduce the significance of any molecular mechanism identified in vitro. To ensure experimental reproducibility, particular care should be taken in the choice of culture conditions: aspects such as cell strains, culture serum, lipoproteins concentration, and culture passages should be carefully considered and explicated in the presentation of results. We aimed to review in vitro studies on mitotane effects, highlighting how different experimental conditions might contribute to the controversial findings. If the concerns pointed out in this review will be overcome, the new insights into mitotane mechanism of action observed in-vitro could allow the identification of novel pharmacological molecular pathways to be used to implement personalized therapy.

## 1. Introduction

Mitotane, 1,1-(o,p′-Dichlorodiphenyl)-2,2-dichloroethane (o,p′-DDD), commercially available as Lysodren^®^ (HRA Pharma Rare Diseases, Paris, France), is a parent compound of the insecticide dichlorodiphenyltrichloroethane (DDT). o,p′-DDD is metabolized by the mitochondria of adrenal cells in DDE (1,1-(o,p′-Dichlorodiphenyl)-2,2 dichloroethene) and DDA (1,1-(o,p′-Dichlorodiphenyl) acetic acid) through α-hydroxylation and β-hydroxylation, respectively. In addition, the unstable precursor of DDA, o,p′-dichlorodiphenyl acyl chloride (DDAC), obtained through cytochrome P540 (CYP450), could covalently bind to mitochondrial macromolecules of adrenal cells or can be metabolized by CYP2B6 in the liver or intestine, reducing its bioavailability [1]. Mitotane is the reference drug for the treatment of advanced adrenocortical carcinoma (ACC) either alone or in combination with chemotherapy [2,3] and is increasingly used for postoperative adjuvant therapy [1,2,3,4,5].

Although mitotane can exert its effects on the gonads and pituitary gland [6,7,8,9], it acts primarily on the adrenal cortex leading to cell destruction and impairment of steroidogenesis [10,11,12]. Indeed, mitotane produces dose-related cellular toxicity causing the rupture of mitochondrial membranes mainly on the zona fasciculata and reticularis, whereas a minimal effect on the zona glomerulosa has been observed [13]. This differential action explains why aldosterone secretion is less affected by mitotane treatment [14,15]. It is generally accepted that circulating levels of mitotane should be maintained between 14 and 20 mg/L (approximately 40–60 µM), the therapeutic window, to obtain the anti-tumoral effect while avoiding severe neurological toxicity [3,16]. Indeed, several retrospective analyzes have shown that mitotane blood concentrations ≥14 mg/L are associated with a disease response in both advanced and adjuvant ACC treatment [17,18,19,20,21,22]. The upper limits are more uncertain; in fact, central neurological toxicity has been more frequently associated with elevated mitotane concentrations (>20 mg/L), but mild symptoms can be observed even with lower plasma levels [17,23]. Studies, however, have suggested that inhibition of steroid secretion could be obtained even with lower mitotane levels [24,25]. Mitotane accumulates in lipoproteins and is stored in adipose tissue, although little is known about how this distribution affects its effectiveness [26]. 

Nevertheless, the mechanism of action of mitotane remains poorly defined at a molecular level due to controversial results generated by in vitro studies addressing its anticancer effect. Here, we will review these in vitro studies on mitotane action highlighting how different experimental conditions might contribute to the controversial results. Further elucidation of mitotane action after a reappraisal of the in vitro experimental conditions may contribute to the implementation of patient-tailored treatment.

## 2. In Vitro Cell Models of ACC

The need to develop appropriate cell models that mimic adrenal physiology or pathology has led to the development of different immortalized ACC cell lines because several issues have limited the use of primary adrenal cells as in vitro models. The most common limitations were (1) the need for fresh tissue, (2) the difficulty in isolating a sufficient number of cells with the adrenocortical phenotype, (3) the difficulty in identifying the cancerous lesions as either primary tumors or metastases from other organs, and (4) the great variability in clones obtained from different human donors, which make their comparison difficult. The variability of primary adrenal cells in terms of drug resistance, hormone production, and gene and protein expression has also recently been reported by van Koetsveld et al. [27]. To overcome these problems, many groups have attempted to establish cell lines from human ACCs, as previously reviewed by Tao Wang and William E. Rainey [28]. For this scope, cells derived from human ACCs were subsequently amplified in vitro with culture media supplemented with different serum additives. For the “in vitro” anti-cancer drugs’ analysis, particularly for studies on mitotane, the most widely used cell models included H295-derived cell strains and SW13 cell lines. 

In particular, the H295 cell line was established from a female patient with ACC whose tumor was extracted, defragmented, and maintained in culture media for one year [29]. The selected cells, called NCI-H295, appear to act as pluripotent adrenal cells capable of producing each of the zone-specific steroids [28]. The parental H295 has a poorly adherent phenotype and a relatively long population doubling time. To address this issue, alternative culture conditions and different commercial sera (Nu-Serum^TM^ type 1, Ultroser^TM^, and Cosmic Calf^TM^ serum) were used to generate three H295R sub-strains. In comparison to the original H295 cell line, the H295R sub-strains showed a tightly adherent phenotype and a reduction in doubling time from five to two days [30]. Cell strains, culture medium, and passaging have a critical impact on the cellular response, growth rate, and steroid production [31,32]. Furthermore, the angiotensin II limited responder strain, H295A, was obtained with a similar strategy, removing nonattached cells during passaging. The H295 progenitor cell line produces more glucocorticoids compared with the H295R and H295A sub-strains, which produce more androgens and mineralocorticoids, respectively [28,31]. Furthermore, in 2008, it was demonstrated, by the SNP array analysis, that the HAC13 and the HAC15 cell lines were not ACC-independent cell models but were monoclonal sub-strains from H295R cells, probably isolated from a sample contaminated with this cell line [33].

The other in vitro human model often utilized in mitotane experiments is the SW13 cell line. These cells were isolated and amplified from a 55-year-old female with a small cell type carcinoma excised from the adrenal cortex [34]. Given their unusual histology and lack of steroidogenic potential, it is unclear whether SW13 cell lines are primary adrenocortical carcinoma or resulting from adrenal cortex metastases [28]. This latter scenario is also supported by studies showing that the SW13 cell model, unlike H295R cells, is responsive to a drug that is mainly effective on lung metastases [35]. Interestingly, mitotane does not appear to be effective on tumor cell lines that originated from the lung [36]. Despite the controversy about the SW13 origin, this cell line has often been used in studies on mitotane as the archetype of a mitotane-resistant cell line.

Recently, to increase the availability of ACC cell models in vitro, some protocols have been developed to extract cells from in vivo patient-derived tumor xenografts (PDTXs). PDTXs have been established for a wide range of cancer types maintaining the original tumor characteristics. However, these tumors often have low growth capacity, limiting the applicability of PDTXs in preclinical studies. This derived cell models could be useful to overtake this limitation [37,38]. The first adult ACC PDTX and the corresponding cell line MUC-1 were recently developed from a 24-year-old male patient with supraclavicular ACC metastasis by Hantel et al. MUC-1 cells maintain hormonal activity in vitro and, even after several passages, the specific phenotypic characteristics for ACC. Furthermore, MUC-1 cells appear to be resistant to routine drug treatment [37]. With a similar approach, Kiseljak-Vassiliades et al. generated two independent ACC cell models: CU-ACC1 and CU-ACC2 [38]. The CU-ACC1 models were derived from a 66-year-old patient who initially presented hypertension and hypokalemia, whereas CU-ACC2 models were developed by liver metastases from a 26-year-old patient with Lynch syndrome. CU-ACC1 and CU-ACC2 share some peculiar characteristics of progenitor tumors. In particular, CU-ACC1 possess a mutation in exon 3 of *CTNNB1* gene despite the allele frequency being higher than both patient-derived tumor and PDTX [38]. CU-ACC2 shares with the PDTX and the patient tumor a deletion of exons 1–6 in *MSH2* gene, which is a deletion often associated with Lynch syndrome [38].

All available ACC cell lines, in animals or humans, show a loss of function of the p53 protein. In particular, a large homozygous deletion of exons 8 and 9 in the *TP53* gene has been identified in cellular strains derived from H295, while a single nucleotide variant that alters the *TP53* coding sequence has been observed in SW13 [39]. MUC1 carry a frameshift deletion of one guanidine on *TP53* gene [37], while p.G245S protein mutation has been identified in CU-ACC2. Although its functional significance has not yet been elucidated, it could affect p53 DNA binding, which has also been reported in other adrenocortical carcinoma samples [38]. In contrast, mutations in *TP53* gene have not been identified in CU-ACC1, despite the drastically reduced p53 protein expression compared to the CU-ACC2 cell line [38]. This situation could partly explain the peculiar cell model characteristics, such as a reduction in corticosteroid production, an altered gene expression, and a different cell doubling time, observed by increasing the culture passages. In fact, it is plausible that the accumulation of mutations over time, favored by the p53 functional lack, leads to the development of different cellular subpopulations with altered drug resistance and/or with different steroidogenic potential [40].

## 3. Mitotane Effects on Mitochondrial Membrane and Gene Expression

**Figure 1:**
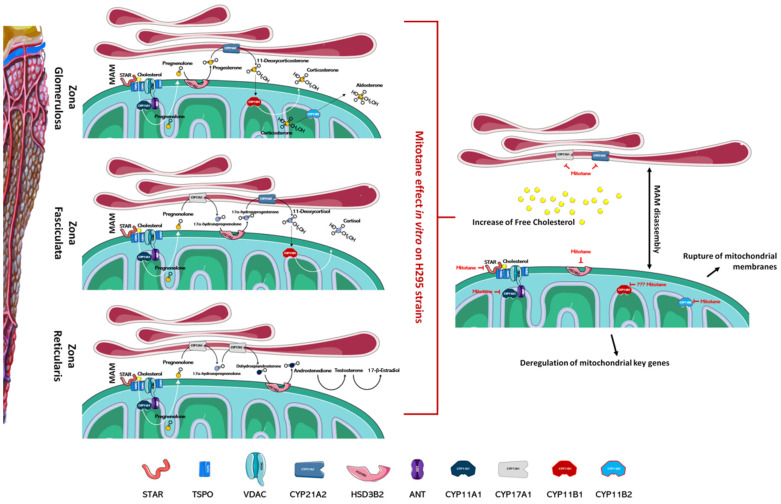
Mitotane impairs the function of the adrenal cortex. In the left part of the figure, the different zones of the adrenal cortex are schematized; the main enzymes involved in the biosynthesis of steroid hormones are also indicated. As depicted in the right part of figure, mitotane action, identified by in vitro experiments, involves several mechanisms ranging from the deregulation of mitochondrial key genes at a transcriptional and functional level, to the MAMs dissociation, the rupture of mitochondrial membranes, and altered cholesterol transports/metabolism. Mitotane action for each enzyme is indicated by a red mark. Figures have been created modifying an image set from Servier Medical Art (SMART) http://smart.servier.com/ (19 July 2021).

Mitotane seems to act selectively on the adrenal cortex affecting steroidogenesis. This specificity for the adrenal cortex could be related to the massive presence in these cells of enzymes involved in steroidogenesis and/or cholesterol metabolism that could interact directly with mitotane (Figure 1). Indeed, mitotane shares characteristics with other endocrine disruptors and may affect steroidogenesis by binding to steroid receptors, mimicking the action of steroids [41]. A binding between mitotane and cytochrome P450 has been directly observed [42,43,44]. Interestingly, this interaction inhibits CYP11A1-mediated metabolic transformation regardless of the presence of the CYP11A1 substrate or its inhibitor. This result may indicate that either CYP11A1 is not the mitotane activator or that mitotane activation is not required to destroy CYP enzyme function. Indeed, the formation of adducts can affect the endogenous function of critical target proteins and thus directly causes toxicity or binds to non-essential proteins and thus constitutes an exposure biomarker [45]. Similar behavior was observed in murine corticosterone-producing Y1 cell line [42]. Furthermore, mitotane-induced protein adducts could also explain the altered transcriptomic profile, with varying degrees of post-translational modifications, identified by Stigliano et al. [12].

Several articles have reported that mitochondria are the organelles primarily involved in mitotane susceptibility in adrenal cells. This action involves several mechanisms ranging from the deregulation of mitochondrial key genes to the rupture of mitochondrial membranes (Figure 1). Mitotane affects mitochondrial enzymes at a transcriptional and functional level and significantly decreases the expression of the protein that transports cholesterol into mitochondria and of its related gene *STAR* [26,31,46]. Inside of mitochondria, cholesterol is converted to pregnenolone by CYP11A1 and, as indicated previously, mitotane mediates functional and transcriptional CYP11A1 inhibition [26,31,46,47,48,49,50]. Further, mitotane-related downregulation of steroidogenic enzymes *HSD3B2*, encoding for 3β-hydroxysteroid dehydrogenase/Δ5-4 isomerase, and *CYP21A2*, encoding for steroid 21-hydroxylase, was also observed [46,51]. Contrasting results were obtained for the *CYP11B1* gene, encoding for the enzyme 11b-hydroxylase, which catalyzes the transformation of 11-deoxycorticosterone and 11-deoxycortisol into corticosterone and cortisol, respectively [31,51,52,53,54]. As for CYP11A1, the CYP11B1 enzyme has also been indicated as an activator of mitotane, but much experimental evidence may suggest that its involvement is not essential in mitotane-induced mitochondrial dysfunction: (1) mitotane interacts with CYP11B1, creating an irreversible bond and decreasing both cortisol and aldosterone secretion in a concentration-dependent manner, yet metyrapone, a known inhibitor of CYP11B1, is unable to modify mitotane-induced effects [1,42]; (2) cells that do not express CYP11B1, or cells that express it, are likewise affected by treatment with mitotane [51]; (3) CYP11B1 modulation in H295R cells, by either chemical or molecular inhibition, is not able to affect mitotane action [54]. At the transcriptional level, depending on the model cell line in the study and/or experimental conditions, *CYP11B1* was observed as either downmodulated [51,53,54] or upmodulated by mitotane treatment [31,52]. To complete the intra-mitochondrial aldosterone synthesis, the enzyme aldosterone synthase, codified by the *CYP11B2* gene, was transcriptionally inhibited by mitotane in vitro [51]. All these enzyme inhibitions, mediated by mitotane, generate mitochondrial dysfunction that correlates with alterations in the ATP/ADP ratio, which is a critical factor to control nuclear gene expression.

SF-1 protein, identified independently by two laboratories in 1992, is the major nuclear factor that determines the cell-specific expression of P450 steroidogenic enzymes in gonads and adrenal glands [55,56]. SF1 activates adenylate cyclase by acting via G protein-coupled receptors, such as ACTH, and thereby increasing cAMP levels. The cAMP response elements (CRE) present in the proximal promoter of all P450 steroidogenic enzymes respond to increased cAMP levels by initiating the synthesis of P450 steroidogenic enzymes. Mitotane blocks the ACTH/cAMP-related signaling, although contrasting results due to specific human cell models have been observed. In particular, H295A are non-responsive, whereas H295R respond to this hormone depending on subclones and culture conditions [28]. The response of the H295 progenitor cell line is not so clear; it is often indicated as ACTH-unresponsive [28] but probably follows the same behavior of H295R cells. Indeed, Lin et al. showed that H295 responds to increasing ACTH concentration by increasing cortisol secretion and that mitotane was able to completely abolish this response [31].

Mitotane could also affect the angiotensin II/K+ related signaling principally responsible for CYP11B2 transcription. All H295R strains, including the subclone HAC15, respond to this molecular signaling pathway, in contrast to H295A, which are selected as not responder cells. No indication of angiotensin II/K+ signaling was obtained for the H295 progenitor cell line [28]. Although all studies agree on the blocking action of mitotane on corticosteroid synthesis, conflicting results in molecular pathways and in the deregulation of specific genes or enzymes could support the hypothesis that specific cell line characteristics and variable experimental conditions have an important impact on mitotane action and should be carefully considered for a meaningful assessment of in vitro studies on mitotane.

## 4. Physiological Regulation of Cholesterol Uptake, Synthesis, and Steroidogenesis and the Proposed Mitotane Effect/Mechanism of Action

**Figure 2:**
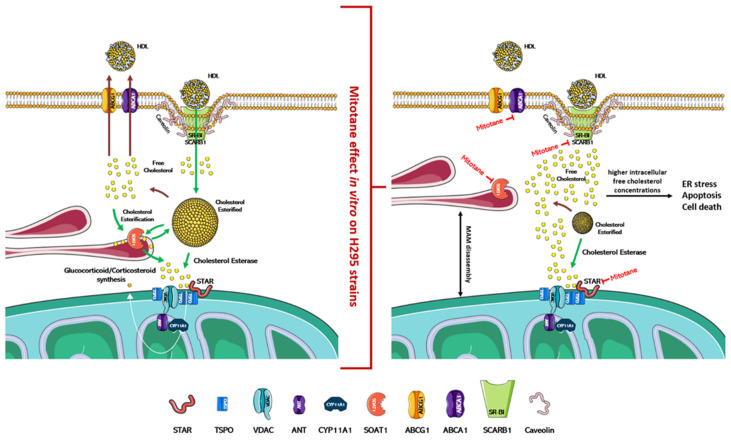
Physiological regulation of cholesterol uptake, synthesis, and steroidogenesis and proposed mitotane effect/mechanism of action. In the left part of the figure is indicated the physiological mechanism that regulates the absorption/synthesis of cholesterol and steroidogenesis. As depicted in the right part of the figure, mitotane induces in vitro the dissociation of MAMs and the blockade of cholesterol transport/synthesis and steroidogenesis. Accumulation of free cholesterol in cells causes ER stress, apoptosis, and cell death. The action of mitotane for each enzyme is indicated by a red mark. Figures were created modifying an image set from SMART http://smart.servier.com/ (19 July 2021).

Mitochondria-associated membranes (MAM) are reversible contact points between the mitochondria and the endoplasmic reticulum (ER) membrane and are involved in the mitochondrial import of certain lipids, such as cholesterol. The presence of several enzymatic targets responsive to mitotane in mitochondria and MAM caused a progressive alteration in mitochondrial structure and the number of normal mitochondria when H295R were exposed to mitotane (Figure 1 and Figure 2). In addition, a more punctiform pattern, as a sign of mitochondrial fragmentation, was frequently observed [51,57]. Further, mitotane exposure alters the MAM integrity, reducing the interactions between mitochondria and ER in H295R [49]. These results could be related to a progressive depolarization of the mitochondrial membrane, also due to the functional block of COX enzymes, with consequent interruption of the respiratory system and MAM disassembly [49,51]. Sterol O-acyltransferase enzymes, SOAT1 and SOAT2, are located within MAM and catalyze cholesteryl esters formation from cholesterol. Sbiera et al. identified SOAT1 as the key molecular target of mitotane and showed a correlation between SOAT1 expression and the outcome of adjuvant mitotane treatment [58], whereas Lacombe et al. found that SOAT1 expression is a prognostic marker in combination with the Ki67 index [59]. Unfortunately, the hypothesis that SOAT1 expression could be a clinically useful marker for predicting treatment response to mitotane has not been confirmed by further studies [27,60]. Weigand et al. retrospectively analyzed data of 231 patients with ACC treated with mitotane in 12 reference centers and did not find any significant differences between tumors with high or low SOAT1 expression in terms of recurrence-free survival (in 158 patients treated with adjuvant mitotane), progression-free survival (in 73 patients with advanced ACC), or disease-specific survival (in both settings) [60].

In vitro, mitotane induces ER stress through inhibition of SOAT1, which leads to the blockade of cholesterol synthesis and steroidogenesis, and this accumulation of free cholesterol rapidly becomes toxic to the cells (Figure 2) [58,61]. Furthermore, mitotane in H295R subclones reduces the expression of ABCA1, which is involved in the cellular efflux of cholesterol [62], and of *SCARB1*, which encodes for scavenger receptor B1 (SR-BI), the most important transporter for adrenal cholesterol uptake [46,63]. The adrenal cortex has critical enzymes and substrates necessary for ferroptosis, a form of iron-dependent cell death associated with increased lipid peroxidation. Curiously, despite the strong induction of lipid peroxidation, mitotane does not induce ferroptosis [64,65]. Since mitotane increases free cholesterol in cells and oxysterols, such as 27-hydroxycholesterol, which could reduce this process [66], the cholesterol metabolism could be an interesting druggable pathway to counteract mitotane resistance in ACC. On these bases, the introduction of LXRα and PCSK9 inhibitors as future therapeutic approaches could be a promising tool to reduce mitotane resistance and/or to optimize its therapeutic dose [46,66]. In the adrenal gland, the role of LXRα and its oxysterol ligands are critically important in the fine regulation of cholesterol efflux since the excess free cholesterol in cells is converted into oxysterols through the action of enzymes, such as CYP27A1. Pharmacological inhibition of LXRα significantly reduces the expression of the cholesterol efflux pump (ABCA1 and ABCG1) and is accompanied by higher intracellular free cholesterol concentrations, ER stress, apoptosis, and cell death markers expression. This effect is complementary to mitotane-induced lipotoxicity, and, using a combined therapeutic approach, lower doses of mitotane can be expected to be used, resulting in reduced toxicity [66].

## 5. Culture Conditions and Mitotane Cytotoxicity: A Need for Reappraisal

The close relationship between cholesterol and mitotane’s chemical structure could also justify the conflicting results obtained in the last decade in evaluating the effect of mitotane in vitro. Since the creation of the original H295 strain, several laboratories have explored the cytotoxic ability of mitotane with mixed success. The IC50 of mitotane, at different time intervals, in the H295 and H295R subclones ranged from the therapeutic dose of about 40–60 μM up to over 100–200 μM (the most relevant experimental conditions are summarized in Table 1). Intriguingly, the work of Hescot et al. seems to throw light on this question by identifying an opposite correlation between the effect mediated by mitotane and the lipoprotein concentration in culture media. In particular, mitotane was more efficient in exerting its toxic effect when cells were grown in a lipoprotein-free medium, indicating that HDL and LDL sequester mitotane, reducing its actions. Furthermore, a similar blocking effect was also observed for bovine serum albumin (BSA) [26]. Lipoproteins and BSA are the most abundant proteins in culture serum, and, except for Lin et al. who used an uncommon medium, there seems to be an opposite relationship between mitotane effect and serum concentration of these proteins in culture media (Table 1). This hypothesis was apparently also confirmed by other authors, who observed that mitotane action was strongly influenced by the culture conditions, the sub-strain selected, and the growth under different serum conditions [32,46,62]. Note that most ACC cell models, such as SW13, MUC1, CU-ACC1, and ACC2, reported in vitro as more resistant to mitotane respect H295 cell strains, which are maintained in high serum/BSA conditions (5–10% FBS) [64,65,66,67]. Intriguingly, mitotane treatment in patients induces hypercholesterolemia via an incompletely understood mechanism that also increases lipoproteins synthesis. This effect is of particular importance as it could potentially self-promote drug resistance [1,26]. On this basis, several in vitro and clinical studies were recently conducted to evaluate how to counteract resistance to mitotane by lowering lipoprotein levels through, for example, statins or PCSK9 inhibitors [61,62,68]. In a recent clinical case, the strategy of targeting the PCSK9 gene [68], which encodes an enzyme expressed mainly in the liver and intestine with an important role in lipid metabolism, was reported. PCSK9 binds to the LDL receptor favoring its degradation with the effect of increasing circulating LDL. Therefore, the inhibition of PCSK9 by monoclonal antibodies leads to an increase in the levels of LDL receptors in the cell surface that bind LDL particles and thus circulating LDL is decreased. Tsakiridou et al. reported the case of a patient with drug-resistant hypercholesterolemia induced by mitotane, in which the administration of evolocumab, a PCSK9 inhibitor, led to a reduction in circulating LDL levels by 36%. This effect allowed to increase the dose of mitotane and to reach therapeutic plasma levels. These data indicate that treatment with PCSK9 inhibitors should be considered in patients who develop mitotane-related hypercholesterolemia that cannot be managed with conventional lipid-lowering treatment [68].

## 6. Conclusions

This review collected several in vitro studies assessing the mechanisms of mitotane action and pointed out the search for new molecular pathways that could define mitotane sensitivity. Mitotane appears to act selectively on the adrenal cortex by influencing steroidogenesis. Several molecular mechanisms have been identified in vitro and involve: deregulation of key mitochondrial genes, such as those encoding the P450 family of cytochromes, both at the transcriptional and functional level; depolarization and rupture of mitochondrial membranes; reduction in interactions between mitochondria and endoplasmic reticulum by altering the integrity of MAMs; reduction in the expression of proteins, such as STAR and SOAT1, involved in cellular uptake and cholesterol metabolism leading to the accumulation of free cholesterol and cell death. The divergent results obtained in presumably identical cell lines highlight the need for a stable in vitro model and/or a standard methodology to perform experiments on H295 strains. To ensure experimental reproducibility, particular care should be given to the choice of culture conditions: aspects such as cell strains, culture serum, lipoproteins and BSA concentration, and culture passages should be carefully considered and explicated in the presentation of results. Specific attention should be paid to the use of fetal bovine serum (FBS) or fetal calf serum (FCS) during cell culture as they represent poorly defined supplements and, therefore, unpredictable experimental variability factors. Indeed, different serum lots show quantitative and qualitative composition variations, and this variability introduces a possible confounder making the experiments difficult to reproduce [72]. In light of these considerations, it might be necessary to re-evaluate the experiments on mitotane to clean them of any confounding factors that could hide important molecular findings. In addition to that, another important aspect to evaluate is the heterogeneity of ACC tumors. This scenario stimulates scientists to create different ACC cell lines to have multiple models resembling variability observed in patients. The concept is fundamental to explain mechanisms of drug resistance that could be subsequentially evaluate in patients; however, it is mandatory that cell line experiments be conducted in a neutral milieu, where only the genetic/molecular characteristics of the model may influence the results, in the absence of other confounding factors. Molecular characterization of ACC achieved using in vitro experiments is a powerful tool that expands knowledge in mitotane molecular mechanism. If these concerns are overcome in future, the new insights into mitotane mechanism of action could allow the identification of novel pharmacological molecular pathways to be used to implement personalized therapy, maximizing the benefit of mitotane treatment and minimizing its toxicity.

## Figures and Tables

**Table 1:** Mitotane cytotoxicity and in vitro culture conditions.

Author	Year	IC50 (μM)	Serum in Experimental Conditions
Chia-Wen Lin [31]	2012	Cell viability not significantly affected by 5–40 μM for 24 h, or 48 μM for 72 h	RPMI1640 supplemented with hydrocortisol (10 pM), β-estradiol (10 pM), no serum in experiments
Poli [57]	2013	10–20 μM (72–48 h)	1% FBS for all the experiments (10% FBS in culture)
Doghman [69]	2013	22.8 μM (144 h)	2% Nu-Serum^TM^
Zsippai [41]	2012	10–100 μM (72–48 h)	2.5% Nu-Serum^TM^
Germano [70]	2015	30.6 μM (72 h)	2.5% Nu-Serum^TM^
Germano [67]	2014	30.62 μM (72 h)	2.5% Nu-Serum^TM^
Sbiera [58]	2015	18.1 μM (24 h)	2.5% FCS (by article doi:10.3389/fendo.2011.00027)
Hescot [26]	2015	40 μM (lipoprotein-free medium) 140 μM (control lipoprotein conditions)	Different experimental conditions [10% FCS in culture]
Hescot [51]	2013	100 μM (45% of cells dead at 48 h)	10% FBS
Hescot [53]	2014	100 μM (48 h) (95% inhibition when treated with 200 and 300 μM)	10% FBS
Boulate [62]	2019	50 μM did not affect cell viability (24–48 h)	10% FBS
Goyzueta Mamani [71]	2021	20–50 μM did not affect cell viability (24 h)	10% FBS
